# Risk of lactic acidosis in type 2 diabetes patients using metformin: A case control study

**DOI:** 10.1371/journal.pone.0196122

**Published:** 2018-05-08

**Authors:** Abdellatif Aharaz, Anton Pottegård, Daniel Pilsgaard Henriksen, Jesper Hallas, Henning Beck-Nielsen, Annmarie Touborg Lassen

**Affiliations:** 1 Department of Endocrinology, Odense University Hospital, Odense, Denmark; 2 Clinical Pharmacology and Pharmacy, University of Southern Denmark, Odense, Denmark; 3 Department of Respiratory Medicine, Odense University Hospital, Odense, Denmark; 4 Department of Emergency Medicine, Odense University Hospital, Odense, Denmark; The University of Tokyo, JAPAN

## Abstract

**Background:**

Metformin constitutes first-line treatment of type 2 diabetes mellitus. It is presumed to have lactic acidosis as a dangerous, but rare, side effect.

**Objectives:**

To estimate the incidence rate of lactic acidosis in patients with type 2 diabetes mellitus as well as to estimate the relative risk of lactic acidosis associated with metformin treatment.

**Methods:**

This is a population-based combined cohort and case-control study among patients with type 2 diabetes mellitus who were acutely admitted with lactic acidosis at Odense University Hospital, Denmark; in the period from 1st June 2009 to 1st October 2013. The patients included as cases were all acutely hospitalized with lactic acidosis (pH <7.35 and lactate ≥2.0 mmol/l). For each case, we identified 24 age- and sex-matched controls sampled from the same cohort with type 2 diabetes mellitus. The use of metformin identified by using a prescription database. Analyses included multivariable logistic regression and adjusting for predefined confounding: renal function, HbA1c, comorbidity and diabetes duration.

**Results:**

Our cohort included 10,652 patients with type 2 diabetes mellitus with a median age of 74 years, and 51.5% were male. During follow-up, 163 individuals were hospitalized with lactic acidosis, corresponding to an incidence rate of 391/100,000 person years. Use of metformin was not associated with lactic acidosis: adjusted odds ratio was 0.79 (95%CI 0.54–1.17).

**Conclusion:**

Among patients with type 2 diabetes mellitus, the incidence rate of acute hospitalization with lactic acidosis was 391/100,000 person years. Use of metformin did not increase the risk of lactic acidosis. However, comorbidity seems to be an important risk factor.

## Introduction

Metformin is first-line treatment of type 2 diabetes mellitus (T2D). There are approximately 380 million diabetes patients worldwide of whom about 120 million use metformin [1–3]. Metformin is presumed to have lactic acidosis (LA) as a dangerous, but rare side effect, although the evidence supporting this association is generally limited [4]. The principal complicating factor is that many clinical conditions may cause LA. The specific risk related to metformin use is therefore difficult to identify among potential confounding factors such as comorbidity, severe infection or dehydration [4–5].

The observed incidence rates of LA among metformin users range from 3-47/100,000 person-years [4,6–10]. However, many studies do not distinguish between LA cases that might be attributed to metformin use and LA cases where competing causes contribute to an elevated lactate level [6,8,10]. A systematic review and pooled analysis of T2D patients from randomized trials and cohort studies found no cases of LA in 70,490 person-years exposed to metformin [11]. Our hypothesis is that metformin is associated with LA and that the wide range of the different incidence rates is imprecise prediction of the number of admissions with this condition.

We aimed to estimate the incidence rate of LA among T2D patients as well as odds ratio of LA associated with the use of metformin in a registry-based study combined with clinical data obtained from acute hospital contacts.

## Methods and materials

### Study design

We conducted a population-based combined cohort and case-control study of T2D patients residing in the catchment area of Odense University Hospital, Denmark. As cases, we included patients hospitalized with LA in the period from 1 June 2009 to 1 October 2013. LA was documented using arterial analyses performed within four hours after arrival to the hospital.

### Setting

The Danish healthcare system is tax-financed and provides its services free of charge to all citizens of Denmark. All acutely ill patients are thus admitted to the nearest public hospital. Odense University Hospital is a tertiary hospital with 1,000 beds, and it also serves as the only primary hospital for a population of 288,000 citizens [12].

**Fig 1:**
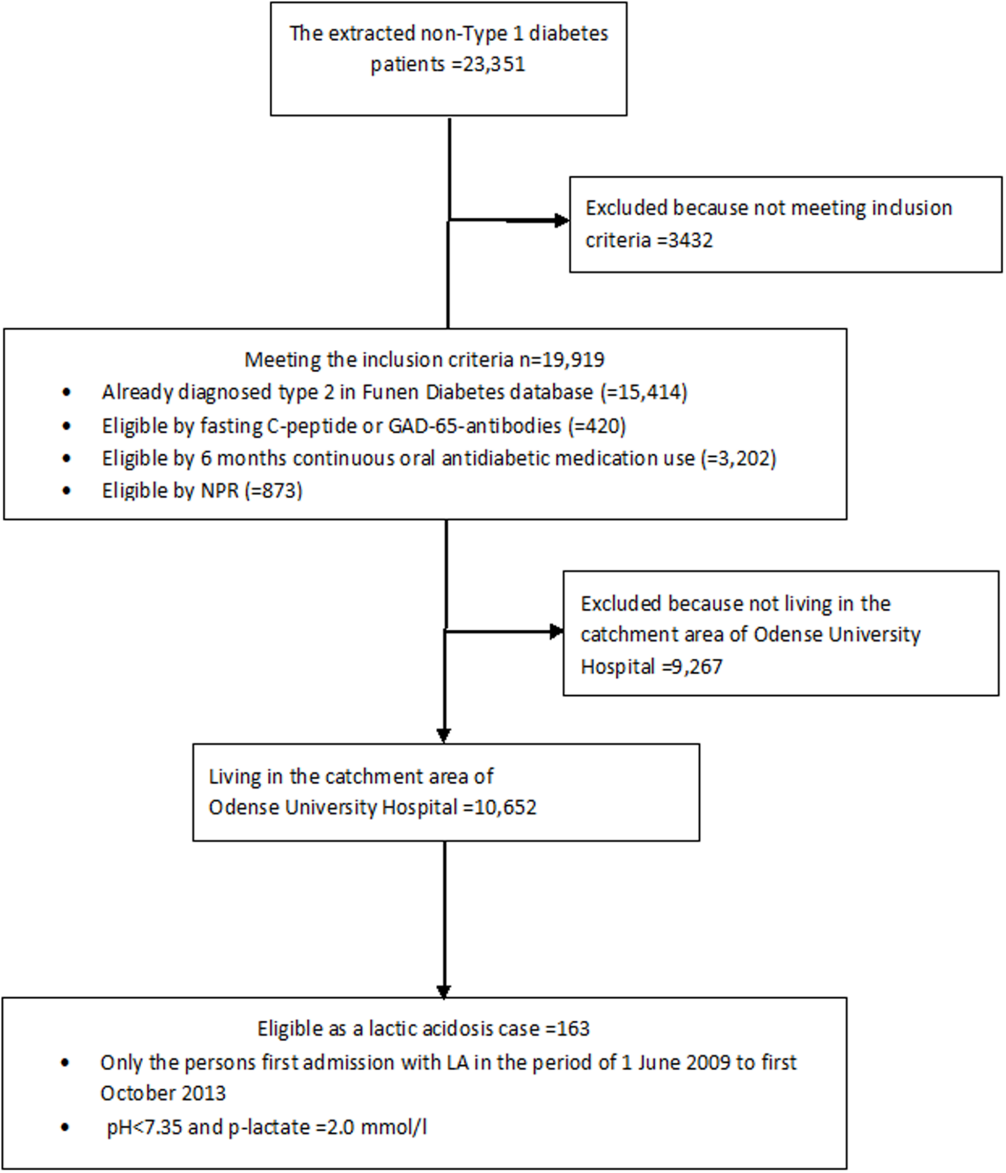
From extraction to cases.

We identified adult T2D patients residing in the catchment area of Odense University Hospital via the Funen Diabetes Database. This clinical database contains clinical and laboratory data of diabetes patients from primary care and from hospital outpatient clinics in a geographical area corresponding to the former Funen County (population 480,000). For patients without a definitive T2D diagnosis recorded in the database, we included the patients as having T2D based on fasting C-peptide, GAD-65-antibodies, continuous use of oral antidiabetic drugs, or a T2D diagnosis recorded in the Danish National Patient Register (for cutoffs and definitions, see (Fig 1)). Patients in the T2D cohort were included for the period they were residing in the catchment area of Odense University Hospital within the period from 1 June 2009 to 1 October 2013.

Patients identified in the T2D cohort were linked to population-based registers using the unique personal identification number assigned to all Danish citizens [13]. For information about medication, we used the Odense Pharmacoepidemiological Database (OPED), which covers the Region of Southern Denmark [14]. It includes redeemed and reimbursed prescriptions from the catchment area of Odense University Hospital since 1990, and uses the hierarchical Anatomical Therapeutic Chemical Classification System (ATC) and the Defined Daily Doses (DDD) developed by the WHO [15]. For discharge diagnosis and comorbidity, we used the Danish National Patient Register. This nationwide register is from 1977 and contains all hospital discharge diagnoses encoded according to ICD-8 from 1977 to 1993 and ICD-10 since 1994 [16].

### Study size

We included all adult type 2 diabetes patients residing in the catchment area of Odense University Hospital who met our eligibility criteria and all available data collected from the data base was used for this analysis.

### Cases

The cases included patients with a first-time hospital admission with LA, documented within four hours after arrival to the hospital by an arterial puncture. We defined LA by a lactate level at or above 2.0 mmol/l and a pH value below 7.35. The date of admission was used as the index date.

We furthermore defined a subgroup with “severe” LA (lactate ≥5.0 mmol/l and pH <7.35) and another subgroup with “idiopathic” LA (lactate ≥2.0 mmol/l and pH <7.35), excluding cases with conditions or use of medications (other than metformin) that might elevate the lactate level (S1 Fig) within seven days before and up to 30 days after admission date. Cases with a cancer diagnosis within the past five years before admission were also excluded from the idiopathic subgroup.

### Controls

Controls were sampled using risk-set sampling from the type 2 diabetes cohort meaning those subjects who met the eligibility criteria for T2D (Fig 1). Thus, all controls had an index date equal to the admission date of their corresponding case and they were matched by sex and year of birth. For each case, we included 24 controls sampled from the cohort of T2D patients.

### Exposure to metformin

We defined a patient’s current use of metformin as having received a metformin prescription dated within 90 days before the index date. A patient having a metformin prescription dated between 90 days and 365 days before the index date was classified as a recent user, while a patient with no prescription for metformin prior the past 365 days prior to sampling was considered as a no-user. A patient who has never received a metformin prescription ever was also considered as a no-user.

### Controlling for confounding

We included the following prespecified confounders in our analysis: renal function, diabetes regulation, Charlson Comorbidity index [17], and diabetes duration. If no diabetes duration was recorded in the Funen Diabetes Database, diabetes duration was defined using the date of the first redeemed antidiabetic medication prescription recorded in OPED.

Until 2011, renal function was given as creatinine. We used the Modification of Diet in Renal Disease (MDRD) formula [18], for calculating the estimated GFR (eGFR): eGFR (ml/min/1.73 m^2^) = 175 × (S_cr_)^-1.154^ × (Age)^-0.203^. If we found several HbA_1c_ values or several eGFR values for a patient within the past 12 months prior to the index date, we calculated the average value of these. Comorbidity was expressed using the Charlson comorbidity index [17]. In the present study, the estimation of the Charlson comorbidity index did not include diabetes nor renal failure, as only diabetes patients were included and as renal failure was adjusted for as an independent covariate in the regression model. Diabetes duration was chosen as a surrogate marker to control for diabetic related complications.

### Patient involvement

Our study is a strictly observational study based on registries. The study design was discussed and chosen by the authors before conducting the study.

### Analyses

The incidence rate of LA was calculated with 95% CI (assuming Poisson distribution). The incidence rate was reported as LA cases per 100,000 person-years among individuals in the T2D patient cohort.

We used multivariable conditional logistic regression for comparison of current use of metformin to non-use of metformin to obtain the OR associating current use of metformin with LA. This analysis was adjusted for eGFR, HbA_1c_, Charlson comorbidity index, and diabetes duration.

Substratum analyses were performed within strata of age, sex, Charlson comorbidity index, renal function, and diabetes duration.

Analyses were carried out using STATA14.1 IC (Statacorp, College Station, TX 77840, USA).

#### Statement of independence of researchers from funders and coauthor approval of the submitted version

There are no commercial funders. The University of Southern Denmark and The Nordic Fund of Bioscience had no role in study design, data collection, data analysis, data interpretation, or writing of the report. All authors were responsible for drafting the article and revising it critically for important intellectual content. All authors approved the version to be published. The manuscript is previously unpublished, and is not submitted simultaneously elsewhere.

### Ethical approval, trail registration and patient involvement

The study was approved by the Danish Data Protection Agency (ID 3027–001) and by the Danish National Board of Health (ID 3-3013-410/1/ GHB). According to Danish law, no ethical approval is needed for registry-based studies [19]. The work complies with the and has been conducted according to internationally accepted ethical standards. Patients, patient lawyers, and patient organizations were not involved in this research.

### Access and sharing of data and materials

Please contact the Research Service at the Department of Clinical Research, University of Southern Denmark, 5000 Odense C. Phone: +45 6550 4051. Up-to-date information on data access is available online:

https://www.sdu.dk/en/Om_SDU/Institutter_centre/Klinisk_Institut/Ledelse_administration/Institutsekretariatet

Access to data from the Danish Health Data Authority requires approval from the Danish Data Protection Agency:

https://www.datatilsynet.dk/english/the-danish-data-protection-agency/introduction-to-the-danish-data-protection-agency/.

The authors do not have special access privileges to these data.

## Results

We identified 10,652 T2D patients that resided in the catchment area of Odense University Hospital and followed them for a total of 41,650 person-years (Fig 1).

Among the 10,652 TD2 patients, 163 experienced an acute hospitalization with LA (lactate ≥2 mmol/l and pH ≤7.35) corresponding to an incidence rate of 391/100,000 person-years (95% CI 334-456/100,000 person-years).

Compared to the 3,834 sex- and age-matched controls, LA cases revealed substantially higher comorbidity rates than controls (Charlson comorbidity score ≥2 in 63.8% of the cases and 24.7% of the controls), while HbA1c and eGFR were comparable (Table 1).

**Table 1:** Characteristics of lactic acidosis cases (lactate ≥2.0 mmol/l and pH <7.35) and matched controls.

Lactate ≥2.0 mmol/l	Cases (n = 163)	Controls (n = 3,834)
**Age**, median (IQR)	74 (66–82)	74 (66–81)
**Gender**		
Male	84 (51.5%)	1,992 (52.0%)
Female	79 (48.5%)	1,842 (48.0%)
**Use of metformin**		
Non-use^a^	78 (47.9%)	1,744 (45.5%)
Recent use^b^	16 (9.8%)	218 (5.7%)
Current use^c^	69 (42.3%)	1,872 (48.8%)
**Diabetes duration**		
0–1 years	11 (6.7%)	424 (11.1%)
2–5 years	34 (20.9%)	705 (18.4%)
6–9 years	25 (15.3%)	492 (12.8%)
10+ year	93 (57.1%)	2,213 (57.7%)
**Charlson comorbidity index**		
0	44 (27.0%)	2,364 (61.7%)
1	15 (9.2%)	523 (13.6%)
≥2	104 (63.8%)	947 (24.7%)
**Laboratory values**		
**eGFR**^d^^,^^e^, mean (SD)	68·4 (32.0)	71.1 (24.2)
**HbA**_**1c**_^f^^,^^g^, mean (SD)	7·0 (1.3)	7.0 (1.1)

^a^. Non-use of metformin is “never use of metformin” or “occurrence of a metformin prescription dated more than 365 days before admission with lactic acidosis”.

^b^. Recent use of metformin is “occurrence of a metformin prescription in the past dated 91 to 365 days before admission with lactic acidosis”.

^c^. Current use is”occurrence of a metformin prescription dated within the past 90 days before admission with lactic acidosis”.

^d^. 14 cases (8.6%) and 799 controls (20.8%) had a missing value for eGFR.

^e^. eGFR calculated by the MDRD fourmula.

^f^. 20 cases (12.3%) and 821 controls (21.4%) had a missing value for HbA_1c_.

^g^. HbA_1c_ (glycated hemoglobin), % of total hemoglobin.

Current use of metformin was not associated with the risk of LA. The crude OR of current metformin use versus non-use was 0.82 (95% CI 0.59–1.16), and the adjusted OR was 0.79 (95% CI 0.54–1.17) (Table 2). Subgroup analysis for all 163 cases, according to sex, age, comorbidity, renal function, and diabetes duration demonstrated similar results (Table 3).

**Table 2:** Crude and adjusted odds ratios for metformin use compared to no-use of metformin associated with lactic acidosis (lactate ≥2.0 mmol/l and pH <7.35).

Metformin use Lactate ≥2.0 mmol/l	Cases	Controls	Crude OR^a^ (95%-CI)	Adjusted OR^b^ (95%-CI)
No-use^c^	78	1,744	1.00 (ref.)	1.00 (ref.)
Recent use^d^	16	218	1.52 (0.85–2.72)	1.17 (0.62–2.21)
Current use^e^	69	1,872	0.82 (0.59–1.16)	0.79 (0.54–1.17)

^a^. Matched by age and sex in a risk-set manner.

^b^. Matched by age and sex and adjusted for Charlson comorbidity index, eGFR, HbA_1c_ and diabetes duration

^c^. No-use of metformin is “never use of metformin or occurrence of a metformin prescription dated more than 365 days before admission with lactic acidosis”.

^d^. Recent use of metformin is “occurrence of a metformin prescription in the past dated 91 to 365 days before admission with lactic acidosis”.

^e^. Current use is “occurrence of a metformin prescription within the past dated 90 days before admission with lactic acidosis”.

**Table 3:** Crude and adjusted odds ratios for current metformin use (independent variable) compared to no-use of metformin associated with lactic acidosis (lactate ≥2.0 mmol/l and pH <7.35) (dependent variable), specified by patient subgroups.

Lactate ≥2.0 mmol/l	Cases: metformin	Cases: no metformin	Controls: metformin	Controls: no metformin	Crude OR^a^ (95%-CI)	Adjusted OR^b^ (95%-CI)
**All**	69	78	1,692	1,591	0.82 (0.59–1.16)	0.79 (0.54–1.17)
**Gender**						
Male	38	35	880	754	0.92 (0.57–1.49)	0.79 (0.46–1.36)
Female	31	43	812	837	0.73 (0.45–1.19)	0.84 (0.48–1.44)
**Age**						
<60 years	9	4	147	135	2.18 (0.63–7.55)	1.55 (0.31–7.73)
≥60 years	60	74	1,545	1,456	0.76 (0.53–1.08)	0.76 (0.51–1.14)
**Charlson comorbidity index**						
0	24	16	1,098	918	1.44 (0.74–2.82)	1.21 (0.56–2.60)
1	7	7	232	221	1.39 (0.29–6.78)	0.75 (0.11–5.01)
≥2	38	55	362	452	0.84 (0.53–1.32)	0.83 (0.50–1.38)
**Kidney function**^c^						
eGFR <30	-	-	6	75	-	-
eGFR 30–90	47	52	1,164	896	0.70 (0.46–1.05)	0.83 (0.53–1.29)
eGFR >90	21	17	522	620	1.49 (0.72–3.09)	1.47 (0.33–6.51)
**Diabetes duration**						
0–5 years	10	15	329	454	1.37 (0.56–3.38)	0.90 (0.17–4.91)
5–10 years	22	20	422	308	0.62 (0.30–1.30)	0.74 (0.28–1.93)
10+ years	37	43	941	829	0.76 (0.47–1.21)	0.87 (0.50–1.51)

^a^. Matched by age and sex in a risk-set manner.

^b^. Matched by age and sex and adjusted for Charlson comorbidity index, eGFR, HbA_1c_ and diabetes duration (the adjusted models exclude the covariate that is analyzed for in that stratum; for instance, in the strata for Charlson, the adjusted models do not include Charlson as a covariate).

^c^. eGFR calculated by the MDRD formula.

We repeated the analysis applying two alternative LA case definitions (“severe” LA subgroup and “idiopathic” LA subgroup). We identified 28 idiopathic LA cases (S1 Table) with a lactate level of ≥2.0 mmol/l and a pH value of <7.35. In this subgroup, the unadjusted and adjusted OR was 1.40 (95% CI 0.63–3.10) and 1.55 (95% CI 0.64–3.72), respectively) (Table 4 and S2 Table). In the analysis of the “severe” LA subgroup (S3 Table) with the case definition of a lactate level of ≥5.0 mmol/l and a pH value of <7.35, we found 34 LA cases. In this subgroup, the unadjusted and adjusted OR was 0.71 (95% CI 0.34–1.52) and 0.73 (95% CI 0.30–1.77), respectively) (Table 5 and S4 Table).

**Table 4:** Crude and adjusted odds ratios for current metformin use compared to no-use of metformin (independent variable) associated with idiopathic lactic acidosis (lactate ≥2.0 mmol/l and pH <7.35) (dependent variable), specified by patient subgroups.

Idiopathic LA, Lactate ≥2.0 mmol/l	Cases: metformin	Cases: no metformin	Controls: Metformin/	Controls: no metformin	Crude OR^a^ (95%-CI)	Adjusted OR^b^ (95%-CI)
**All**	17	11	307	274	1.40 (0.63–3.10)	1.55 (0.64–3.72)
**Gender**						
Male	13	2	180	140	5.29 (1.16–24.08)	4.90 (1.01–23.73)
Female	4	9	127	134	0.45 (0.13–1.53)	0.74 (0.19–2.91)
**Age**						
<60 years	-	-	26	17	-	-
≥60 years	16	10	281	257	1.48 (0.65–3.40)	1.90 (0.73–4.89)
**Charlson comorbidity index**						
0	9	5	205	166	1.64 (0.52–5.17)	1.32 (0.41–4.20)
1	-	-	49	42	-	-
≥2	6	6	53	66	1.17 (0.32–4.20)	5.31 (0.26–108.13)
**Kidney function**^c^						
eGFR <30	-	-	-	-	-	-
eGFR 30–90	15	7	209	173	1.61 (0.63–4.09)	1.92 (0.73–5.04)
eGFR >90	-	-	96	89	-	-
**Diabetes duration**						
0–5 years	-	-	64	87	-	-
5–10 years	-	-	90	56	1.90 (0.30–12.08)	err.
10+ years	11	8	153	131	1.14 (0.43–3.07)	1.70 (0.48–6.01)

^a^. Matched by age and sex in a risk-set manner.

^b^. Matched by age and sex and adjusted for Charlson comorbidity index, eGFR, HbA_1c_ and diabetes duration (the adjusted models exclude the covariate that is analyzed for in that stratum; for instance, in the strata for Charlson, the adjusted models do not include Charlson as a covariate).

^c^. eGFR calculated by the MDRD formula.

**Table 5:** Crude and adjusted odds ratios for current metformin use compared to no-use of metformin associated with severe lactic acidosis (lactate ≥5.0 mmol/l and pH <7.35), specified by patient subgroups.

Lactate ≥5.0 mmol/l	Cases: metformin	Cases: no metformin	Controls: metformin	Controls: no metformin	Crude OR^a^ (95%-CI)	Adjusted OR^b^ (95%-CI)
**All**	13	17	352	331	0.71 (0.34–1.52)	0.73 (0.30–1.77)
**Gender**						
Male	9	8	209	176	0.95 (0.36–2.55)	1.03 (0.29–3.69)
Female	4	9	143	155	0.46 (0.13–1.61)	0.49 (0.13–1.85)
**Age**						
<60 years						
≥60 years	13	17	352	331	0.71 (0.34–1.52)	0.73 (0.30–1.77)
**Charlson comorbidity index**						
0	7	5	219	187	1.00 (0.31–3.27)	0.82 (0.22–3.12)
1	-	-	54	51	-	-
≥2	5	11	79	93	0.67 (0.20–2.21)	0.42 (0.09–1.88)
**Kidney function**^c^						
eGFR <30	-	-	3	15	-	-
eGFR 30–90	7	13	257	205	0.39 (0.15–1.02)	0.43 (0.15–1.26)
eGFR >90	-	-	92	111	2.04 (0.35–11.86)	err.
**Diabetes duration**						
0–5 years	-	-	70	76	-	-
5–10 years	5	5	77	91	1.21 (0.32–4.63)	4.54 (0.28–74.52)
10+ years	6	8	205	164	0.52 (0.17–1.60)	0.55 (0.14–2.16)

^a^. Matched by age and sex in a risk-set manner.

^b^. Matched by age and sex and adjusted for Charlson comorbidity index, eGFR, HbA_1c_ and diabetes duration (the adjusted models exclude the covariate that is analyzed for in that stratum; for instance, in the strata for Charlson, the adjusted models do not include Charlson as a covariate).

^c^. eGFR calculated by the MDRD formula.

## Discussion

We found that the incidence rate of LA among T2D patients was 391/100,000 person-years. However, irrespective of case definition, we found no increased risk of acute hospitalization with LA associated with metformin use for T2D patients.

Although, metformin is widely presumed to increase the risk of LA, our analyses were unable to confirm this. Our findings are in line with the results of a systematic review of T2D patients including 347 comparative trials and cohort studies, covering 70,490 metformin person-years and 55,451 non-metformin person-years, which found no cases of LA in the metformin group and in the comparison group [11]. Our results are also in line with the LA incidence rates among metformin users found in several cohort studies [4,6,8]. On the other hand, our findings contrast with other observational studies that describe an increased risk of LA among metformin-treated diabetes patients compared to diabetes patients without metformin treatment [9,10,20]. We made a post-study power calculation based on a case/control ratio of 1:24, and based on a metformin exposure of 45% in the control group, a type-1 error of 5% and a power of 90%, resulting in the possibility to detect an odds ratio of 1.7 or higher. The overall adjusted odds ratios in our study did not exceed 1.7 (Tables 3, 4 and 5).

Until recently, there was no consensus about the cutoff levels defining LA, but most recent studies have used the following levels: lactate ≥5 mmol/l and pH <7.35 [7,21–24]. In one study, the cutoff levels were pH <7.20 and lactate >5 mmol/l [25], while another study used pH <7.0 and lactate >10 mmol/l [26].

We defined LA as the non-physiological values of pH and lactate, meaning pH <7.35 and lactate ≥2 mmol/l. We chose to include a “severe” LA subgroup (lactate ≥5 mmol/l) to ensure comparability between our study and previous studies. Importantly, irrespective of the LA definitions used, we found no increased risk of LA among metformin users. Not surprisingly, the incidence rate of LA depends on the definition of LA. In the present study, the incidence rate of LA in T2D patients was 391 LA cases/100,000 person-years (95% CI 334–456) for all LA cases with a lactate level of ≥2 mmol/l. For “idiopathic” LA cases, the rate was 67/100,000 person-years (95% CI 45–97). For all LA cases with a lactate level of ≥5 mmol/l, the rate is 82/100,000 person-years (95% CI 57–114).

Some of the studies assessing the LA incidence among metformin-treated T2D patients used case definitions based on discharge diagnosis or on patient records mentioning LA. In contrast, the present study identified cases based on the lactate level and the pH value measured at the patient’s arrival to the hospital. Case identification based on registers or patient records might present a risk of registration bias, as clinicians who know the putative link between metformin and LA might be more prone to give a patient a diagnosis of LA if the patient is treated with metformin. In the present study, the cases were identified by an arterial puncture performed at the patient’s arrival to the hospital. These tests were performed prospectively and were based on the patient´s clinical presentation often without prior knowledge of any antidiabetic treatment received by the patient. Lastly, underreporting in registry-based/patient-recorded-based studies might explain why our LA incidence estimates are higher than in most other studies.

In our study, we found that LA cases showed considerably higher comorbidity rates than T2D controls (Table 1 and S5 Table). Another study has reported similar results [27]. However, until now, the systematic evaluation of the impact of comorbidity has focused on specific comorbidity groups such as renal failure or heart failure [9,27–29]. A Portuguese study showed that renal function is an independent risk factor in elevated lactate concentrations [20], which is also a trend suggested in our study in our unexplained subgroup, but is not shown as a significant result. A group in Michigan, United States, also found an association between elevated lactate levels and increased comorbidity in T2D patients with normal renal function [30]. We found a wide variety of causes of LA but the two main groups were respiratory diseases and infections including septicemia.

### Strengths and limitations

The major strength of our study is the population-based design and the well-defined cohort of T2D patients. A further strength is our case identification, which is based on the patient’s clinical presentation at arrival to the hospital, unrelated to the patient’s use of metformin.

About 45% of hospitalized acute medical patients at Odense University Hospital have an arterial puncture performed at arrival [31]. The decision to perform an arterial puncture is based on the physician’s clinical judgement. As all patients with even minor clinical deteoriation have an arterial puncture performed in the present setting. We believe, that all—or nearly all—acute medical patients clinically affected by LA are identified. However, if patients present with lactate acidosis—but no clinical symptoms they are not included in the analysis. In parallel, patients dead before arrival to hospital or immediately after arrival where no arterial puncture is performed are not included in the study.

As the subgroup analyses were exploratory and the analyses were underpowered given the number of cases identified and the number of variables included in the model all results should be interpreted with this in mind.

As a limitation, our study is a single-hospital study, and as such it does not necessarily represent other hospitals. However, Odense University Hospital serves as the only primary hospital for a well-defined population, and we therefore believe that our results can be generalized to acute settings in other primary hospitals.

## Conclusion

The incidence rate of acute LA in T2D patients is 391 per 100,000 person-years, and the risk of acute hospitalization with LA was unrelated to the use of metformin. However, comorbidity seems to be an important risk factor, but this needs evaluation in further studies.

## Supporting information

S1 Fig ICD-10 for Lactate-Elevating conditions (Conditions, comorbidities, drugs and medications. (DOCX)

S1 Table Characteristics of idiopathic lactic acidosis cases (lactate ≥2.0 mmol/l and pH <7.35) and matched controls. (DOCX)

S2 Table Crude and adjusted odds ratios for metformin use associated with idiopathic lactic acidosis (lactate ≥2.0 mmol/l and pH <7.35). (DOCX)

S3 Table Characteristics of lactic acidosis cases (lactate ≥5.0 mmol/l and pH <7.35) and matched controls. (DOCX)

S4 Table Crude and adjusted odds ratios for metformin use associated with lactic acidosis (with lactate ≥5.0 mmol/l and pH <7.35). (DOCX)

S5 Table Comorbidity in cases and in controls. (DOCX)
