# Supplementary material for: Risk of lactic acidosis in type 2 diabetes patients using metformin: A case control study
Source: PLoS One. 2018 May 8;13(5):e0196122. doi: 10.1371/journal.pone.0196122 (PMC5940216; doi:10.1371/journal.pone.0196122)
Supplement: S1 Fig — (DOCX) [file pone.0196122.s001.docx]

**S1 Fig2**. ICD-10 for lactate-Elevating Conditions (Conditions, comorbidities, drugs and medications.

**Postcardiac arrest, Mesenteric ischemia, Burns, Trauma, Compartment syndrome, Diabetic ketoacidosis, Heavy exercise, Excessive work of breathing, Thiamine deficiency**

(DI46 DI469 DO7291 DO742B DO754E DO754F DO891B DK551F DT20)

(DF1001 DF1101 DF1201 DF1301 DF1401 DF1501 DF1601 DF1701 DF1801)

(DF1901 DZ916 DM622A DT796E DM726 DE101 DE111 DE121 DE131 DE141)

(DT782A DT788A DR060 DR061 DR062 DR063 DR064 DR068 DR068A DR068C)

(DE51 DE511 DE511A DE511B DE512 DE518 DE519)

**Drugs and medical**

(DT398A EUXR6005 EUYR6005 MN02BE01 MN02BE51 MN02BG71)

(DF10 DZ721) exclude(DF1021 DF1022 DF1023 DF1026 DF106 DF107)

(MR03AA MA01AD01 MB02BC09 MC01CA24 MR01AA14 MR03AA01 MR03AK01)

(MS01EA01 MS01EA51 NAAD52A NAAD52B) (DF140) (DT650) (DT523) (DT511) (MJ05AB)

**Seizures**

(DF1006 DF1031 DF1041 DF1106 DF1131 DF1141 DF1206 DF1306 DF1331)

(DF1341 DF1406 DF1431 DF1506 DF1531 DF1606 DF1706 DF1806 DF1906)

(DF1931 DF1941 DF445) (DG402 DG403 DG408 DG409)

(DR252 DR252A DR56 DR560 DR560A DR568 DR568C DR568D DR568E)

**Liver**

(DC70 DC71 DC72 DC73 DC74 DK752 DK753 DK754 DK754A DK758)

(DK759 DK759A DK76 DK77) exclude (DK768E)

**Respiratory**

(DJ40 DJ41 DJ44 DJ45 DJ46 DJ60 DJ61 DJ62 DJ63 DJ64 DJ668)

(DJ67 DJ68 DJ70 DJ80 DJ81 DJ84 DJ90 DJ91 DJ93 DJ94 DJ95)

(DJ96 DJ98 DJ99) exclude (DJ980A DJ980B DJ980C)

**Shock minus septic shock**

(DR57 DT63 DT634F DT886 DT780 DT780A DT782 DT782A DT788A DT811) (DT754A DO083 DO751 DT794 DA483)

**Infections**

(DA390 DA392A DA869 DA870 DA879 DB003 DB004 DB020)

(DB022 DB022A DB022B DB919 DG001 DG008 DG009 DG009A DG019 DG040)

(DG042 DG060 DG060F DG062 DG079)

(DA150 DA151 DA152 DA159 DA310A DA481 DB371 DB909 DJ120 DJ139 DJ42)

(DJ149 DJ15 DJ150 DJ151 DJ152 DJ154 DJ155 DJ157 DJ158 DJ159 DJ69)

(DJ170 DJ178C DJ18 DJ180 DJ181 DJ188 DJ189 DJ209 DJ209A DJ219 DJ229)

(DJ409 DJ440 DJ690 DJ698 DJ698A DJ851 DJ852 DJ860 DJ869)

(DA419B DN109 DN129 DN136 DN300 DN308 DN309 DN390 DN390B)

(DA009 DA011 DA020 DA038 DA043 DA045 DA047 DA048 DA049 DA542 DA059)

(DA080 DA081 DA082 DA083 DA084 DA085 DA09 DA099 DB670 DK350 DK350A)

(DK351 DK351A DK352 DK353 DK359 DK570 DK572B DK573 DK573A DK573B)

(DK573F DK579A DK650 DK650A DK650G DK650J DK650M DK650N DK650O)

(DK658 DK659 DK658I DK659 DK670 DK671 DK672 DK673 DK750 DK803 DK804)

(DK810 DK819 DK830 K861B DN733 DN734 DN735)

(DI300 DI301 DI308 DI309 DI330 DI339 DI389 DI398)

(DA469 DB001A DB001B DB372 DK610 DK610A DK611 DK612 DL022 DL022T)

(DL024 DL024F DL024K DL029 DL029A DL031 DL031E DL033 DL088 DL089)

(DM000 DM002 DM002A DM008 DM009 DM463 DM464 DM465 DM465A DM469)

(DM711 DM861 DM868 DM869)

(DA515 DA799 DB001 DB059 DB204 DB206 DB208 DB230 DB232 DB249 DB258)

(DB259 DB270 DB279 DB509 DB529 DB549 DB550 DB589 DJ091 DJ099 DJ100)

(DJ108 DJ11 DJ110 DJ111 DJ118)

(DA329 DA401 DA403 DA408 DA409 DA410 DA411 DA411A DA412 DA413 DA414)

(DA415 DA418 DA419 DA419A DA429 DA449 DA482 DA490 DA491 DA493 DA498)

(DA499 DA499A DA689 DA709 DA812 DB008 DB029 DB340 DB349 DB369 DB370)

(DB377 DB378 DB379 DB809 DB899 DB955 DB956 DB956A DB964 DB965 DB968)

(DB999 DM726 DR50 DR500 DR508 DR509 DT814D DT846 DT899)

(DB002A DB023G DB373A DB374 DB378C DE060 DE061 DH651 DH660 DH669)

(DJ009B DJ010 DJ011 DJ012 DJ018 DJ019 DJ020 DJ029 DJ029B DJ030 DJ039)

(DJ039A DJ040 DJ051 DJ069 DJ369 DJ390C DK040A DK053A DK102C DK112C)

(DK121 DK628L DN412 DN450B DN459 DN459A DN764A DO868)

**Septic shock**

(DA41 DA410 DA411 DA411A DA412 DA413 DA414 DA415 DA418 DA419 DA419B)

(DA419C DT880 DO753)

**Cancer minus malignant melanoma**

(DC) ex (DC42)
