# Supplementary material for: Risk of lactic acidosis in type 2 diabetes patients using metformin: A case control study
Source: PLoS One. 2018 May 8;13(5):e0196122. doi: 10.1371/journal.pone.0196122 (PMC5940216; doi:10.1371/journal.pone.0196122)
Supplement: S1 Table — (DOCX) [file pone.0196122.s002.docx]

**S1 Table 6.** Characteristics of idiopathic lactic acidosis cases (lactate ≥2.0 mmol/l and pH <7.35) and matched controls

| **Idiopathic LA,**  **Lactate ≥2.0**  **mmol/l** | **Cases** | **Controls** |
| --- | --- | --- |
| **All** | (n=28) | (n=622) |
| **Age**, median (IQR) | 73 (66 - 81) | 72 (65 - 81) |
| **Gender** |  |  |
| Male | 15 (53.6%) | 334 (53.7%) |
| Female | 13 (46.4%) | 288 (46.3%) |
| **Use of metformin** |  |  |
| Non-use^a^ | 11 (39.3%) | 274 (44.1%) |
| Recent use^b^ | 0 (0.0%) | 41 (6.6%) |
| Current use^c^ | 17 (60.7%) | 307 (49.4%) |
| **Diabetes duration** | |  |
| 0-1 years | 1 (3.6%) | 67 (10.8%) |
| 2-5 years | 5 (17.9%) | 110 (17.7%) |
| 6-9 years | 3 (10.7%) | 88 (14.1%) |
| 10+ year | 19 (67.9%) | 357 (57.4%) |
| **Charlson comorbidity index** |  |  |
| 0 | 14 (50.0%) | 397 (63.8%) |
| 1 | 2 (7.1%) | 96 (15.4%) |
| ≥2 | 12 (42.9%) | 129 (20.7%) |
| **Laboratory values** |  |  |
| **eGFR**^d,e^, mean (SD) | 60·8 (26.1) | 70·0 (22.6) |
| **HbA_1c_**^g,f^, mean (SD) | 7·0 (1.2) | 7·0 (1.2) |

a. Non-use of metformin is “never use of metformin or occurrence of a metformin prescription dated more than 365 days before admission with lactic acidosis”.

b. Recent use of metformin is “occurrence of a metformin prescription in the past dated 91 to 365 days before admission with lactic acidosis”.

c. Current use is “occurrence of a metformin prescription dated within the past 90 days before admission with lactic acidosis”.

d. Two cases (7.1%) and 111 controls (17.8%) had a missing value for eGFR.

e. eGFR calculated by the MDRD formula.

f. Two cases (7.1%) and 108 controls (17.4%) had a missing value for HbA_1c_.

g. HbA_1c_ (glycated hemoglobin), % of total hemoglobin.
