# Supplementary material for: Risk of lactic acidosis in type 2 diabetes patients using metformin: A case control study
Source: PLoS One. 2018 May 8;13(5):e0196122. doi: 10.1371/journal.pone.0196122 (PMC5940216; doi:10.1371/journal.pone.0196122)
Supplement: S2 Table — (DOCX) [file pone.0196122.s003.docx]

**S2 Table 7:** Crude and adjusted odds ratios for metformin use associated with idiopathic lactic acidosis (lactate ≥2.0 mmol/l and pH <7.35)

| **Metformin use Lactate ≥2.0**  **mmol/l** | **Cases** | **Controls** | **Crude OR^a^**  **(95%-CI)** | **Adjusted OR^b^**  **(95%-CI)** |
| --- | --- | --- | --- | --- |
| Non-use^c^ | 11 | 274 | 1.00 (ref.) | 1.00 (ref.) |
| Recent use^d^ | (n<5) | 41 | (-) | (-) |
| Current use^e^ | 17 | 307 | 1.40 (0.63-3.10) | 1.55 (0.64-3.72) |

a. Matched by age and sex in a risk-set manner.

b. Matched by age and sex and adjusted for Charlson comorbidity index, eGFR, HbA_1c_ and diabetes duration.

c. No-use of metformin is “never use of metformin or occurrence of a metformin prescription dated more than 365 days before admission with lactic acidosis”.

d. Recent use of metformin is “occurrence of a metformin prescription in the past dated 91 to 365 days before admission with lactic acidosis”.

e. Current use is “occurrence of a metformin prescription dated within the past 90 days before admission with lactic acidosis”.
