# Supplementary material for: Risk of lactic acidosis in type 2 diabetes patients using metformin: A case control study
Source: PLoS One. 2018 May 8;13(5):e0196122. doi: 10.1371/journal.pone.0196122 (PMC5940216; doi:10.1371/journal.pone.0196122)
Supplement: S3 Table — (DOCX) [file pone.0196122.s004.docx]

**S3 Table 8.** Characteristics of severe lactic acidosis cases (lactate ≥5.0 mmol/l and pH <7.35) and matched controls

| **Lactate ≥5.0**  **mmol/l** | **Cases** | **Controls** |
| --- | --- | --- |
| **All** | (n=34) | (n=810) |
| **Age**, median (IQR) | 74 (70 - 83) | 74 (70 - 83) |
| **Gender** |  |  |
| Male | 21 (61.8%) | 498 (61.5%) |
| Female | 13 (38.2%) | 312 (38.5%) |
| **Use of metformin** |  |  |
| Non-use^a^ | 17 (50.0%) | 373 (46.0%) |
| Recent use^b^ | 4 (11.8%) | 43 (5.3%) |
| Current use^c^ | 13 (38.2%) | 394 (48.6%) |
| **Diabetes duration** | |  |
| 0-1 years | 3 (8.8%) | 71 (8.8%) |
| 2-5 years | 11 (32.4%) | 156 (19.3%) |
| 6-9 years | 4 (11.8%) | 111 (13.7%) |
| 10+ year | 16 (47.1%) | 472 (58.3%) |
| **Charlson comorbidity index** |  |  |
| 0 | 12 (35.3%) | 490 (60.5%) |
| 1 | 3 (8.8%) | 120 (14.8%) |
| ≥2 | 19 (55.9%) | 200 (24.7%) |
| **Laboratory values** |  |  |
| **eGFR**^d,e^, mean (SD) | 65.2 (26.8) | 69·0 (22.7) |
| **HbA_1c_**^f.g^, mean (SD) | 6.8 (0.8) | 6·9 (1.1) |

a. Non-use of metformin is “never use of metformin or occurrence of a metformin prescription dated more than 365 days before admission with lactic acidosis”.

b. Recent use of metformin is “occurrence of a metformin prescription in the past dated 91 to 365 days before admission with lactic acidosis”.

c. Current use is “occurrence of a metformin prescription within the past dated 90 days before admission with lactic acidosis”.

d. Three cases (8.8%) and 161 controls (19.9%) had a missing value for eGFR.

e. eGFR calculated by the MDRD fourmula.

f. Five cases (14.7%) and 157 controls (19.4%) had a missing value for HbA_1c_.

g. HbA_1c_ (glycated hemoglobin), % of total hemoglobin.
