# Supplementary material for: Risk of lactic acidosis in type 2 diabetes patients using metformin: A case control study
Source: PLoS One. 2018 May 8;13(5):e0196122. doi: 10.1371/journal.pone.0196122 (PMC5940216; doi:10.1371/journal.pone.0196122)
Supplement: S4 Table — (DOCX) [file pone.0196122.s005.docx]

**S4** **Table 9.** Crude and adjusted odds ratios for metformin use associated with severe lactic acidosis (with lactate ≥5.0 mmol/l and pH <7.35)

| **Metformin use Lactate ≥5.0**  **mmol/l** | **Cases** | **Controls** | **Crude OR^a^**  **(95%-CI)** | **Adjusted OR^b^**  **(95%-CI)** |
| --- | --- | --- | --- | --- |
| Non-use^c^ | 17 | 373 | 1.00 (ref.) | 1.00 (ref.) |
| Recent use^d^ | (n<5) | 43 | (-) | (-) |
| Current use^e^ | 13 | 394 | 0.71 (0.34-1.52) | 0.73 (0.30-1.77) |

a. Matched by age and sex in a risk-set manner.

b. Matched by age and sex and adjusted for Charlson comorbidity index, eGFR, HbA_1c_ and diabetes duration.

c. No-use of metformin is “never use of metformin or occurrence of a metformin prescription dated more than 365 days before admission with lactic acidosis”.

d. Recent use of metformin is “occurrence of a metformin prescription in the past dated 91 to 365 days before admission with lactic acidosis”.

e. Current use is ”occurrence of a metformin prescription dated within the past 90 days before admission with lactic acidosis”.
