# Supplementary material for: Risk of lactic acidosis in type 2 diabetes patients using metformin: A case control study
Source: PLoS One. 2018 May 8;13(5):e0196122. doi: 10.1371/journal.pone.0196122 (PMC5940216; doi:10.1371/journal.pone.0196122)
Supplement: S5 Table — (DOCX) [file pone.0196122.s006.docx]

**S5 Table 10.** Comorbidity in cases and in controls.

| Charlson Comorbidity Index components | Cases | Controls |
| --- | --- | --- |
| AMI | 19 (11.7%) | 253 (6.6%) |
| Heart failure | 47 (28.8%) | 262 (6.8%) |
| Peripheral vascular disease | 19 (11.7%) | 155 (4.0%) |
| Cerebrovascular disease | 35 (21.5%) | 441 (11.5%) |
| Dementia | 63 (1.6%) | 63 (1.6%) |
| Hemiplegia | <5 | 5 (0.1%) |
| Pulmonary disease | 60 (36.8%) | 273 (7.1%) |
| Rheumatic disease | <5 | 78 (2.0%) |
| Peptic ulcer | 12 (7.4%) | 112 (2.9%) |
| Liver disease, mild | 6 (3.7%) | 20 (0.5%) |
| Diabetes complication | 74 (45.4%) | 521 (13.6%) |
| Leukemia | <5 | 17 (0.4%) |
| Lymfoma | <5 | 24 (0.6%) |
| Solid tumors | 20 (12.3%) | 270 (7.0%) |
| Liver disease, severe | <5 | 13 (0.3%) |
| Solid tumors metastasis | <5 | 29 (0.8%) |
| HIV / AIDS | 0 (0.0%) | 0 (0.0%) |
